# Effects of a Transtheoretical Model–Based mHealth Intervention on Transition Readiness in Adolescents With Epilepsy: Quasi-Experimental Study

**DOI:** 10.2196/70085

**Published:** 2025-11-11

**Authors:** Qing Xia, Shuangzi Li, Ting Wang, Mingping Fan, Jie Xia, Lingling Xie, Huaying Yin

**Affiliations:** 1 Department of Neurology Children’s Hospital of Chongqing Medical University National Clinical Research Center for Child Health and Disorders, Ministry of Education Key Laboratory of Child Development and Disorders, Chongqing Key Laboratory of Child Neurodevelopment and Cognitive Disorders Chongqing China; 2 Department of Child Health Care Children’s Hospital of Chongqing Medical University National Clinical Research Center for Child Health and Disorders, Ministry of Education Key Laboratory of Child Development and Disorders, Chongqing Key Laboratory of Child Neurodevelopment and Cognitive Disorders Chongqing China

**Keywords:** epilepsy, adolescence, transitional services, trans-theoretical model, mobile health

## Abstract

**Background:**

Enhancing self-management and transition readiness in adolescents with epilepsy is essential for successful transition to adult care. The combination of the transtheoretical model (TTM) and mobile health (mHealth) management provides a framework for reducing intervention costs while personalizing care.

**Objective:**

This quasi-experimental study evaluates the feasibility of TTM-based mHealth management for improving transition services in adolescents with epilepsy.

**Methods:**

A total of 98 adolescent patients with epilepsy aged 12-18 years were recruited. Using a nonrandomized design based on treatment locations, they were allocated into either the intervention group (n=49) or the control group (n=49). The intervention group received a TTM-based mHealth management program, which included phase-specific group sessions led by a multidisciplinary team and conducted via Tencent Meeting every 2 weeks or monthly (biweekly for the precontemplation, contemplation, and preparation, and monthly for the action and maintenance). The sessions involved lectures, discussions, and a mini-program that provided disease management support, motivational strategies, and digital reminders tailored to each stage. The control group received conventional remote extended care, consisting of biweekly group lectures and discussions for all patients and their families via Tencent Meeting, supplemented by regular health education materials delivered through a WeChat group. Telephone follow-ups were conducted at the third and sixth months. The total intervention duration was 6 months for both groups. Outcomes were assessed after 6 months using the self-management stage, Self-Management and Transition to Adulthood with Rx=Treatment questionnaire, and a self-developed program acceptability questionnaire.

**Results:**

Postintervention, the intervention group demonstrated significantly better self-management behavior stages compared with controls. At the end of 6 months of intervention, the majority of participants in the intervention group reached the action stage (16/49, 32.65%) and maintenance stage (14/49, 28.57%), whereas most controls remained in precontemplation (12/49, 24.49%) and contemplation stages (13/49, 26.53%). Both groups showed significant improvements from baseline in medication management, health care participation, disease knowledge, doctor-patient communication, and transition readiness total scores at 6-month follow-up (all *P*<.05). Notably, the intervention group achieved additional incremental benefits versus controls (medication management: 3.81, 95% CI 1.26-6.36; health care engagement: 2.77, 95% CI 0.52-5.02; disease knowledge: 1.30, 95% CI 0.28-2.31; provider communication: 3.42, 95% CI 1.62-5.22; transition readiness: 11.30, 95% CI 5.70-6.89; effect sizes [Cohen *d*] ranged from 0.527 to 0.864, indicating moderate-to-large clinical effects). The overall satisfaction scores were 4.43 (SD 0.50) for patients and 4.16 (SD 0.82) for health care providers.

**Conclusions:**

The TTM-based mHealth management program may effectively improve self-management behavior changes and enhance readiness for transition among adolescents with epilepsy, thereby facilitating a smooth transition to adult health care. The program demonstrated high acceptability, providing a reference for establishing clinical transition service protocols. However, this study was a single-center, quasi-experimental trial with a small sample size and short intervention duration. The findings need to be confirmed by larger-scale randomized controlled trials to verify efficacy.

## Introduction

Epilepsy is a common chronic childhood disorder, and approximately 50% of children with epilepsy have seizures that continue into adulthood [1]. This subset of children with epilepsy will need to be transitioned to adult medical care, and experts recommend that the medical transition should begin in early adolescence (11-15 years of age) and extend through to adulthood [2]. If the health care transition for adolescents to adults with epilepsy does not go well, it may lead to treatment interruption–induced seizures, exacerbation [3], emotional problems [4], and social problems such as lack of education and social isolation [5]. Transition readiness is one of the important elements influencing the successful health care transition of chronically ill adolescents [6], and is the ability of chronically ill adolescents to begin to complete the transition process with their families and health care support systems [7]. Early preparation for transition, communicating transition information to chronically ill adolescents, being patient-centered, and assisting patients in acquiring self-management knowledge and skills are important principles to follow when providing transition services [2,8]. Ideal transition service plans must encourage patients to take control of their lives [5]. Therefore, developing self-management behaviors and improving transition readiness in adolescents with epilepsy are critical steps in successful medical transition.

Currently, there are various intervention measures for self-management of patients with epilepsy, and the outcome indicators are highly heterogeneous [9]. Among common intervention methods, group-based interventions have broader coverage and can reduce costs, but they often fail to adequately account for individual differences. In contrast, personalized interventions focus on addressing individual needs but require greater human and time resources, which face constraints due to China’s current limited medical resources [10]. Therefore, how to reduce intervention costs while addressing personalized needs remains an unresolved challenge.

The transtheoretical model (TTM) is a staged model for promoting behavioral change, and the TTM emphasizes the use of stage-specific behavior change strategies tailored to individuals at different stages, while prioritizing the development of self-efficacy to facilitate active engagement in behavioral modification [11], and previous studies have demonstrated the model’s positive significance in the transformation of personal health behaviors in patients with diabetes, chronic kidney disease, and cardiovascular disease [12-14], that provides a theoretical basis for personalized collective intervention from the behavioral stage. In addition, the mobile health (mHealth) management model has been widely noticed and explored in recent years [15,16], with notable applications in self-management for patients with epilepsy and transition care for adolescents with epilepsy transitioning to adulthood [17-19], demonstrating promising potential in epilepsy health management. mHealth’s quick and convenient features can reduce labor costs and time spent on medical care, providing a new perspective for adolescents with epilepsy to achieve transitional services. This study aims to develop an intervention program based on the TTM and the mHealth model, followed by empirical validation. It is anticipated that this program will improve self-management behaviors and transition readiness among adolescents with epilepsy, while also gaining acceptance from both patients and health care providers. The details are reported as follows.

## Methods

### Study Design

This quasi-experimental study with a 2-arm cluster allocation design was conducted and reported in accordance with the Transparent Reporting of Evaluations with Nonrandomized Designs statement [20].

### Ethical Considerations

This study was approved by the Ethics Committee of Children’s Hospital of Chongqing Medical University (approval number: 2021, Ethical Review [Research] number 146). All procedures involving human participants were conducted in accordance with the ethical standards of the institutional and national research committees and with the 1964 Helsinki declaration and its later amendments or comparable ethical standards. As the study involved adolescents with epilepsy and the collection of their medical and questionnaire data, a full ethical review was mandatory and obtained prior to the commencement of the study. Written informed consent was obtained from all parents or legal guardians of the participating adolescents. Additionally, written assent was provided by all adolescent participants themselves. They were thoroughly informed about the study’s purpose, procedures, potential risks and benefits, and their right to withdraw from the study at any time without any detriment to their medical care. For the secondary analysis of data generated within the study’s own mini-program, the original informed consent covered the use of this data for research purposes, as confirmed by the ethics committee, thereby waiving the need for additional consent for analysis. All participant data were anonymized and deidentified prior to analysis. Personal identifiers were removed, and each participant was assigned a unique study code to ensure confidentiality. Data were stored on encrypted, password-protected servers with access restricted to the principal investigator and authorized research personnel only. The mini-program development company signed a legally binding patient information confidentiality agreement to ensure the protection of all participant data. No financial compensation was provided to participants. However, all participants in both groups received the standard care and additional remote support (either the TTM-based program or conventional extended care) at no cost throughout the study period. This included access to educational materials, remote consultations, and continuous support from the health care team.

### Objects of Study

Adolescents with epilepsy (12-18 years old) who visited the outpatient department of a tertiary children’s specialty hospital in Chongqing between August 2021 and February 2022 were screened for eligibility. Inclusion criteria were diagnosis of nonrefractory epilepsy for 6 months or more and seizure-free for 30 days; epilepsy diagnostic criteria were the 2014 International League Against Epilepsy’s definition of epilepsy [21], and categorization criteria were the revised version of the 2017 International League Against Epilepsy [22]; exclusion criteria were (1) impaired use of smartphones, (2) communication disorders, and (3) comorbidities with other systemic chronic or malignant diseases or psychiatric disorders. Written informed consent and parental assent were obtained.

### Sample Size Calculation

An a priori sample size calculation was performed using G*Power software (version 3.1.9.7; Heinrich-Heine-University Düsseldorf). The calculation was based on the primary outcome of transition readiness, as measured by the total score of the Chinese version of the Self-Management and Transition to Adulthood with Rx=Treatment (STARx) questionnaire [23]. The sample size was estimated using the formula (
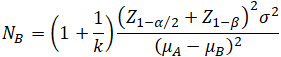
). The parameters for the calculation were specified as follows: a 2-tailed significance level (α) of 0.05, a statistical power (1-β) of 0.90, and an allocation ratio (intervention: control) of 1:1 (ie, k=1). The effect size (Cohen *d*) was set at 0.714. This effect size was derived from a pilot study we conducted prior to the main trial, which included 15 adolescents with epilepsy in each group (30 total). The pilot results indicated a mean difference of 5.0 points in the change of STARx total scores between the intervention and control groups, with a pooled SD of 7.0 points (d=5.0/7.0≈0.714), which represents a moderate-to-large effect. Based on these parameters, the power analysis indicated that a minimum of 42 participants per group was required. To account for a potential dropout rate of 10% during the 6-month intervention period, the target sample size was increased to 49 participants per group, resulting in a total required sample of 98 participants.

### Clinical Data Collection

The clinical data collected encompassed the following categories.

#### Baseline Demographic and Clinical Characteristics

These included age, sex, duration of epilepsy, current antiepileptic medications, seizure frequency in the past 6 months, and any comorbid conditions. This information was primarily collected on-site during the initial enrollment interview using a standardized case report form.

#### Primary and Secondary Outcome Measures

This referred to the data from the validated questionnaires, which were collected using Questionnaire Star at both baseline (T0) and the 6-month follow-up (T1). The specific measures included the following: the primary outcome was the total score and domain scores (medication management, health-care engagement, disease knowledge, and provider communication) of the Chinese version of the STARx questionnaire [23], while the secondary outcomes were the self-management behavior stage assessment form and the self-developed program acceptability questionnaire (for the intervention group only at T1).

#### Process Data (For the Intervention Group)

Data generated within the mini-program, such as medication adherence records (from reminder logs), self-recorded seizure frequency and mood diaries, and engagement metrics (eg, frequency of accessing educational materials), were automatically collected by the platform. A combination of on-site (for baseline characteristics) and Questionnaire Star (for outcome measures) was used for data collection. All data were organized using Microsoft Excel software and were independently checked by 2 researchers to ensure accuracy.

### Evaluation Indicators

#### Stages of Self-Management Behavior

This study incorporates terminological modifications to the definitions of the behavioral stages in conjunction with the TTM to ensure that the meanings of the scale questions are clear. Self-management behaviors in adolescents with epilepsy are listed according to the comprehensive definition of “self-management” in children with epilepsy by Wagner et al [9]. Finally, the assessment form was finalized after expert consultation. That is, “Based on the self-management performance listed above, have you started to self-manage?” patients were asked to select the most appropriate answer, including the preintentional (“never thought about self-management or thought about it 6 months later”), the intentional (“didn’t start to self-manage, but I started to think about it and am going to start in 6 months”), the preparatory (“didn’t start to self-manage, but I’m going to start in 30 days”), the action (“I’ve already started to self-manage, but in less than 6 months”), and the maintenance (“I have been self-managing for more than 6 months”). In order from the preintentional, intentional, preparation, action to the maintenance, the closer the individual is to the maintenance of behavior, the more conducive to successful behavior change. This self-management behavior stage assessment form is an investigator-developed instrument, and its design strictly followed scientific standards: (1) taking the TTM theory as the core framework, and selecting assessment dimensions based on the definition of self-management in children with epilepsy proposed by Wagner et al [9]; (2) inviting a panel of 3 chief physicians of child neurology, 2 pediatric nursing experts, and 2 psychology professors to conduct content validity testing on the assessment items, with the expert content validity index reaching 0.92; (3) a pilot test was conducted on 20 adolescents meeting the inclusion criteria before the formal study, and the results showed that the Kappa coefficient of the assessment form was 0.83 (*P*<.001), indicating good interrater reliability.

#### Transition Preparation

The STARx questionnaire was used, which was originally developed by American scholars Ferris et al [23] to measure chronic disease. Adolescent self-management and readiness for health care transition were validated among adolescents aged 12 to 25 years with chronic illnesses and showed adequate internal reliability, with a Cronbach α coefficient of 0.80 for the questionnaire. The research team from the School of Nursing at Shanghai Jiao Tong University translated and culturally adapted the questionnaire. The Chinese version of the STARx questionnaire demonstrated good internal consistency, with a Cronbach α coefficient of 0.812. The instrument comprises 4 domains as follows: medication management, health care engagement, disease knowledge, and provider communication, totaling 18 items [24]. For scoring, medication-related items (with the option “I am not currently taking medications” scored as 6 points) range from 1 to 6 points. All other items are scored from 1 to 5 points. Higher total scores indicate better transition readiness levels.

#### Acceptance of the Intervention Program in the Experimental Group

Participating study health care professionals and adolescents with epilepsy in the experimental group were given a questionnaire at the end of the intervention to assess the acceptability of the program, which was a self-administered questionnaire with appropriate questions and scores on the operability, usefulness, convenience, affordability, and clinical feasibility of the program. The questionnaire used a Likert 5-point scale with “5” being strongly agree and “1” being strongly disagree. This intervention program acceptability questionnaire is an investigator-developed instrument, and its development process was as follows: (1) core dimensions affecting intervention acceptability (operability, usefulness, and convenience) were identified through literature review; (2) initial items were designed with reference to published medical intervention acceptability assessment tools (eg, the mHealth intervention satisfaction questionnaire developed by Yamamoto et al [25]), resulting in 15 candidate items; (3) 3 ambiguous items were deleted after review by the aforementioned expert panel, and 12 formal items were finally determined; (4) pilot test results showed that the overall Cronbach α coefficient of the questionnaire was 0.84, and the Cronbach α coefficients of each dimension ranged from 0.78 to 0.86, indicating good internal consistency reliability.

### Intervention Methods

#### Experimental Group Intervention Program

Based on the literature review, the research team developed a draft intervention protocol, which was finalized through expert consultation and pilot testing.

#### Self-Management Applet Design and Development

The design team includes 2 medical and nursing specialists, each in child neurology, and one counselor II. The R&D team is a Chengdu Technology Limited Liability company. The applet is used to help adolescents with epilepsy recognize the disease and manage it, and it is set up with a patient side and a health care side. Functions on the patient’s side include learning about epilepsy and self-management, medication reminders, epilepsy logs (based on the pediatric version recommended by the Chinese Association Against Epilepsy, including mood, medication, and seizure records), and data export, as well as patient-physician communication and interaction. Functions on the provider’s side include managing patients’ information, disseminating health education knowledge, viewing patients’ epilepsy logs, and patient-physician communication and interaction. Prior to formal use, adolescents with epilepsy were selected to try out the program until their ease of use, utility, and satisfaction with the applet were all 80% or above. Before the official use of the mini-program, a patient information confidentiality agreement was signed with the mini-program development company.

#### Intervention Implementation Process

**Figure 1:**
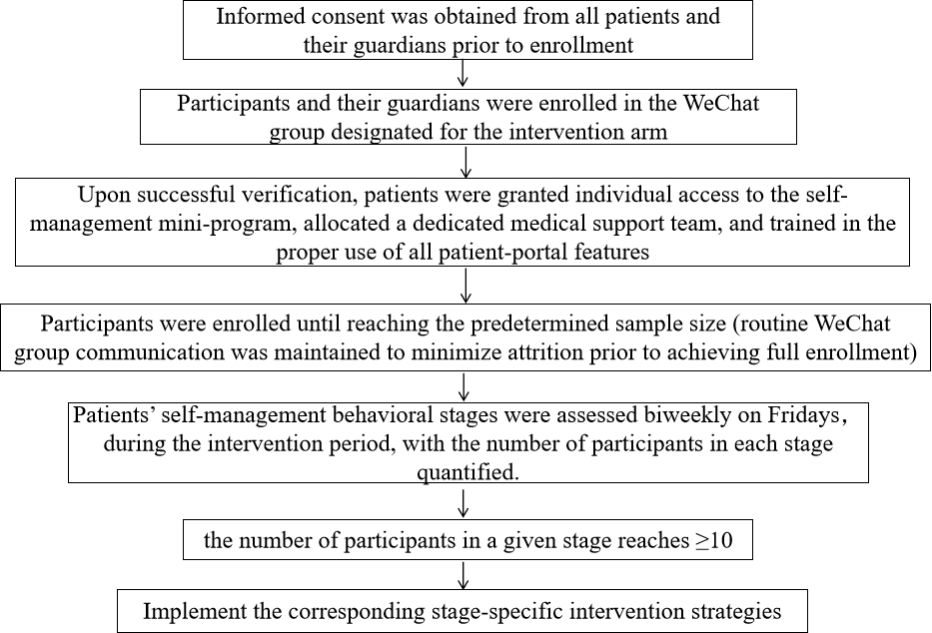
Intervention implementation flowchart.

The intervention lasted for 6 months (24 weeks) and was conducted through a phased, group-based online intervention combining a WeChat mini-program, WeChat, and Tencent Meeting. The implementation process involved 3 main stages. (1) Explain the research purpose and protocol to patients and their guardians. After obtaining written informed consent, patients and parents join the WeChat group for the experimental group. (2) Patients individually access the mini-program to complete their personal information, and then patients are assigned a medical team consisting of “one doctor, one nurse, and one psychological counselor.” This assigned medical team was subsequently responsible for delivering the phase-specific group interventions to their assigned patients. The team explains the mini-program’s functions and use, including how to access health education materials and log medication details, seizure records, and mood diaries in the mini-program. Patients can leave messages in the communication area for special circumstances. (3) Every 2 weeks (on Friday), each patient’s behavioral stage is assessed. When the number of patients in a particular stage reaches ≥10, the corresponding stage-based intervention strategy is implemented, with both patients and parents participating. Since individuals in the action and maintenance demonstrate higher levels of information, motivation, and behavioral skills [11], the intervention frequency is reduced from biweekly (for other stages) to monthly for these 2 stages. The implementation process is illustrated in Figure 1.

#### Implementation Strategy

The mHealth intervention program, based on the TTM, was delivered in stages through group sessions held every 2 weeks or monthly (biweekly for the precontemplation, contemplation, and preparation; monthly for the action and maintenance). These sessions included structured lectures and discussions conducted via Tencent Meeting, combined with a mini-program that provided stage-appropriate disease management support, motivational strategies, and automated reminders. The group sessions were facilitated by a dedicated multidisciplinary transition team, which included a child neurologist, a pediatric epilepsy specialist nurse, and a psychological counselor. The neurologist and nurse were responsible for delivering the stage-specific educational lectures on topics such as epilepsy pathophysiology, medication adherence, and seizure first-aid. The psychological counselor led the motivational interviewing and discussion sessions, using techniques tailored to each TTM stage (eg, exploring ambivalence in the contemplation stage, strengthening commitment in the preparation stage). All facilitators received prior training on TTM principles and the intervention protocol to ensure consistency. Details are presented in Table 1.

**Table 1:** Forms of intervention and intervention strategies for different self-management behavioral stages in adolescents with epilepsy. The group lecture and interactive intervention strategy were delivered via Tencent Meeting.

Plateau and forms of intervention		Intervention strategies	Intervention frequency
**Precontemplation**			1 per fortnight
	Group lectures	Lectures were given on the long-term management of epilepsy, so that patients and their families can recognize the treatment and prognosis of epilepsy, and the importance of preparing for the transition from adolescence to adulthood and self-management.	
	Collective exchange	After the lecture, invite 2-3 patients to share their current concerns, and have the speaker address them.	
	Guided learning	The facilitator will guide patients to access the mini-program’s education section to read stage-appropriate articles: “Epilepsy Treatment & Prognosis,” “Long-term Epilepsy Management,” and “Understanding Healthcare Transition.”	
**Contemplation**			1 per fortnight
	Group lectures	The lectures are identical to the preconsciousness.	
	Collective exchange	During the postlecture sharing session, a volunteer parent may discuss 3 caregiving aspects: receiving the diagnosis, managing uncontrolled symptoms, and coping with caregiver dependency. The speaker will present a case example of a patient with excellent self-management from our outpatient follow-up cohort, to enhance participants’ perception of treatment benefits.	
	Guided learning	The facilitator will guide patients to the mini-program’s education section to study the phase-appropriate article “Top 10 Q&A on Epilepsy,” enabling them to acquire accurate health care knowledge and dispel disease-related misconceptions and unnecessary lifestyle restrictions.	
**Preparation**			1 per fortnight
	Group lectures	Conducting lectures on daily safety management, medication management, and follow-up management of patients with epilepsy, so that patients can learn how to properly self-manage and how parents can collaborate, and learn about available medical resources.	
	Collective exchange	The host medical staff guides each patient to share their current preparations for self-management in terms of material and information resources, and to learn from each other.	
	Sign the pledge form	Distribute a self-management e-commitment form and ask each patient to sign it electronically.	
	Prepare a planner	Patients are required to complete a self-management plan form. The session facilitator will collect the submissions and provide guidance for clearly inappropriate plans.	
	Guided learning	The facilitator will guide patients to access the mini-program’s education section to read stage-specific health articles: “Emergency Seizure Management at Home,” “Medication Adherence Guidelines,” “Optimal Follow-up Practices,” and “Preparing for Adult Healthcare Transition.”	
**Action**			1 per month
	Group lectures	Lecture: Continuing Management of Chronic Diseases in the Information Age, so that patients and parents can master the management of diseases with the help of information technology, such as internet hospitals, medication management applets, and so on.	
	Collective exchange	The facilitator will guide patients and parents to articulate challenges and barriers encountered during implementation, followed by group discussions to develop solutions, enabling mutual learning and experience sharing. The facilitator will specifically emphasize reducing parental over-involvement in patient affairs and encourage independent decision-making to enhance the patient’s self-efficacy.	
	Record in the epilepsy diary	All patients are required to complete an epilepsy diary in the mini-program (including seizure episodes, medication intake, and mood records) at least once weekly. Health care providers will review the submitted seizure logs, medication adherence, and mood entries every Friday, with timely interventions for any recording errors or concerning mood indicators.	
	Guided learning	The facilitator will guide patients to access the mini-program’s education section to read phase-specific articles: “When Should I Transition to Adult Healthcare?” and “How to Be Fully Prepared for Transition?”	
	Enable reminders	The mini-program activated medication reminder alerts to reinforce patient adherence behaviors.	
	Engage reward	A reward mechanism is established where patients earn stars for completing health education modules or epilepsy diary entries at preset frequencies. Accumulated stars can be redeemed for material rewards upon reaching specified thresholds.	
**Maintenance**			1 per month
	Group lectures	Conducting lectures on the mental health and social adaptation of adolescents with epilepsy; in addition, collecting opinions from parents to conduct targeted lectures.	
	Collective exchange	The presiding medical staff guided each patient to share the good methods and problems encountered in the operation, so that we could learn from each other.	
	Enable reminders	Same action.	
	Engage reward	Same action.	
	Guided learning	The facilitator will guide patients to review the stage-appropriate educational article “Coping with Emotional Regulation Challenges” in the mini-program’s learning section. Subsequent push notifications will be tailored based on real-time feedback from patients and parents regarding emerging concerns.	

### Control Group Intervention Program

The control group used conventional continuing care management, instructed patients to record paper epilepsy logs, and established a WeChat group to facilitate communication. After reaching the predetermined sample size, collective lectures will be conducted via Tencent Meeting for all patients and their parents on Sundays of the 2nd, 4th, 6th, 8th, and 10th weeks postenrollment. The lectures will follow the content sequence of the intervention protocol for the experimental group, covering stages from precontemplation to maintenance. Each lecture will be followed by a discussion and questions and answers session. Additionally, one educational article per week will be uploaded to the WeChat group for patients and parents to study. These articles will align with the educational push notifications from the mini-program corresponding to the experimental group’s stages (from precontemplation to maintenance). Telephone follow-ups will be conducted at the 3rd and 6th months.

### Reporting Guidelines

The authors have completed the Transparent Reporting of Evaluations with Nonrandomized Designs checklist for reporting of nonrandomized trials. The completed checklist is available as Multimedia Appendix 1.

### Statistical Methods

Statistical analysis was performed using SAS (version 9.4; SAS Institute Inc) software. Baseline balance between the experimental and control groups was assessed using standardized mean differences (SMDs), with an SMD <0.1 indicating good balance. Categorical variables were described using counts and percentages, and comparisons between groups were conducted using the chi-square test. The normality of continuous variables was evaluated using quantile-quantile plots. Normally distributed continuous variables were expressed as mean (SD), and between-group comparisons were performed using *t* tests. The between-group differences, 95% CIs, and Cohen *d* were calculated using analysis of covariance adjusted for baseline covariates to describe effect sizes. For ordinal categorical variables, between-group comparisons were conducted using the Mann-Whitney *U* test. For stage-transition ordinal data, between-group differences were analyzed using an ordinal logistic regression model adjusted for baseline covariates, with odds ratios (ORs) and 95% CIs reported to describe effect sizes. Within-group prepost changes were assessed using the Wilcoxon signed-rank test. A 2-sided *P* value <.05 was considered statistically significant.

## Results

### General Information

**Figure 2:**
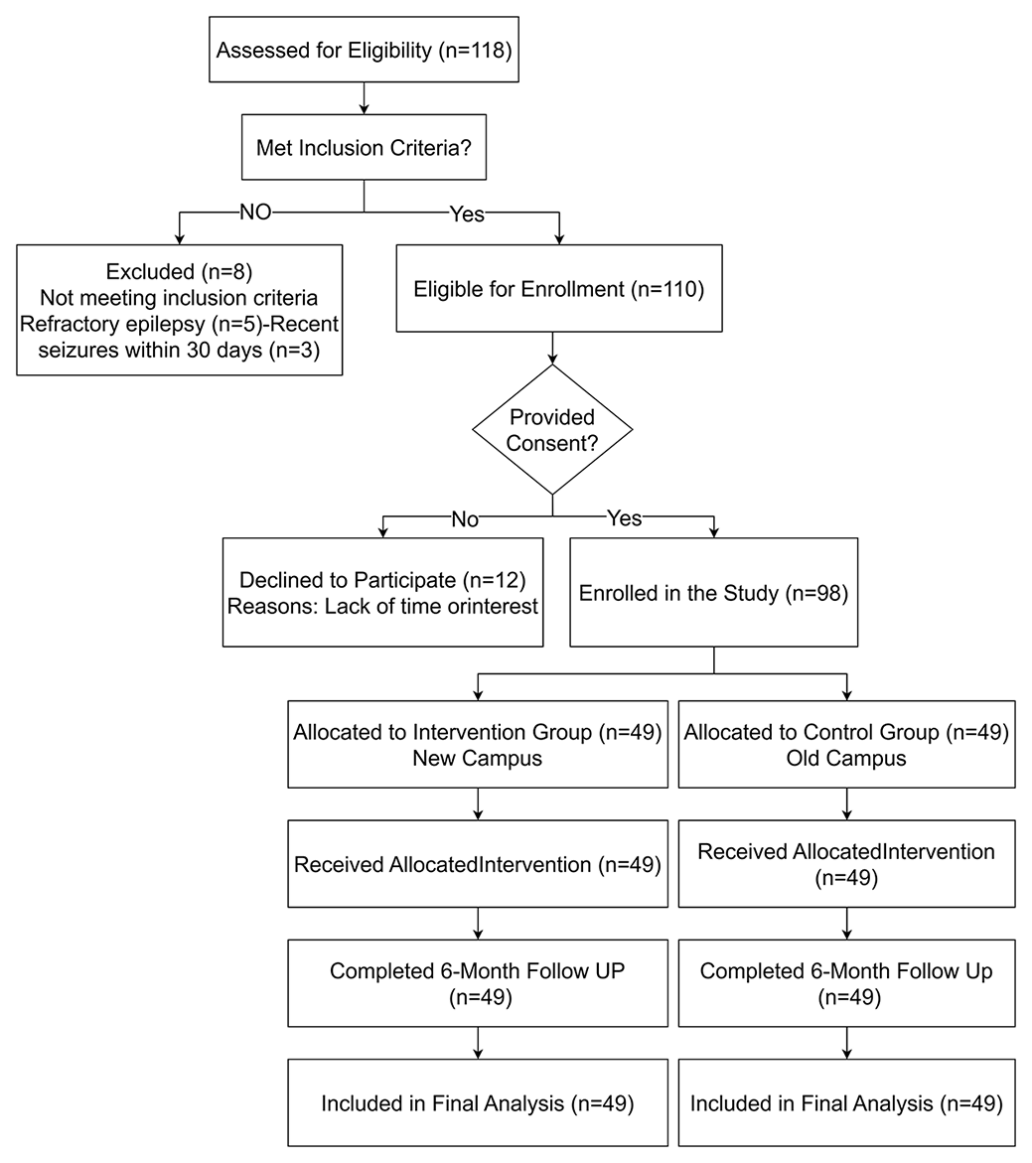
Flow diagram of participant enrollment, allocation, and analysis.

A total of 118 adolescents were assessed for eligibility during the study period. Of these, 20 were excluded: 8 did not meet the inclusion criteria (5 due to refractory epilepsy, 3 due to recent seizures within 30 days), and 12 declined to participate (citing reasons such as lack of time or interest). The remaining 98 eligible adolescents who provided written informed consent (and parental assent) were enrolled in the study. To avoid contamination between study groups, participants recruited from the new campus were assigned to the intervention group (n=49), while those from the old campus served as the control group (n=49). A flowchart detailing the participant screening, enrollment, and allocation process is provided in Figure 2. In this study, 52 cases were enrolled in the experimental group and 52 cases in the control group, among which 1 case in the experimental group withdrew from the study due to diagnosis of leukemia during the intervention period, and 2 cases voluntarily chose to withdraw from the study in the middle of the study; 2 cases in the control group refused to withdraw from the follow-up visit, and 1 case was lost to the follow-up visit; finally, 49 cases in each of the experimental group and the control group completed the study. The 2 groups showed baseline differences (SMD >0.1) in gender, only-child status, disease duration, caregiver type, caregiver education level, and residence (Table 2).

**Table 2:** Comparison of the general information of the 2 groups of patients.

Projects			Experimental group (n=49)		Control group (n=49)		Chi-square (*df*)			*P* value			Standardized mean difference	
**Sex, n (%)**								1.475 (1)			.23			0.247
	Male	20 (40.8)		26 (53.1)										
	Female	29 (59.2)		23 (46.9)										
**Age (years), n (%)**								0.044 (1)			.83			0.043
	12-15	32 (65.3)		31 (63.3)										
	15.1-18	17 (34.7)		18 (36.7)										
**An only child, n (%)**								1.111 (1)			.29			0.214
	Yes	20 (40.8)		15 (30.6)										
	No	29 (59.2)		34 (69.4)										
**Duration illness (year), n (%)**								1.449 (3)			.65			0.160
	≤1	2 (28.57)		5 (71.43)										
	1-3	12 (50.00)		12 (50.00)										
	3-5	21 (51.22)		20 (47.78)										
	>5	14 (53.85)		12 (46.15)										
**Caregiver, n (%)**								1.576 (2)			.46			0.252
	Oneself	6 (12.2)		5 (10.2)										
	Grandparents	9 (18.4)		5 (10.2)										
	Father/Mother	34 (69.4)		39 (79.6)										
**Caregiver literacy, n (%)**								0.379 (2)			.83			0.112
	Junior high school and below	21 (42.9)		19 (38.8)										
	High school or higher vocational	10 (20.4)		9 (18.4)										
	College and above	18 (36.7)		21 (42.9)										
**Current address, n (%)**								0.378 (2)			.83			0.122
	Township	12 (24.5)		11 (22.4)										
	District and county	20 (40.8)		23 (46.9)										
	Municipal and above	17 (34.7)		15 (30.6)										
**Monthly household income (thousands of dollars), n (%)**								2.895 (3)			.41			0.091
	≤2	3 (6.1)		1 (2.0)										
	2-4	15 (30.6)		17 (34.7)										
	4-8	18 (36.7)		23 (46.9)										
	>8	13 (26.5)		8 (16.3)										

### Comparison of Self-Management Behavioral Stages Between the 2 Groups Before and After the Intervention

There was no statistical difference in the preintervention self-management behavioral stage between the 2 groups (*P*>.05). At the end of 6 months of intervention, the intervention group demonstrated significantly better self-management behavior stages compared with controls (OR 2.543, 95% CI 1.207-5.357, *P*=.01). At the end of 6 months of intervention, the majority of participants in the intervention group reached the action stage (16/49, 32.65%) and maintenance stage (14/49, 28.57%), whereas most controls remained in precontemplation (12/49, 24.49%) and contemplation stages (13/49, 26.53%), as shown in Table 3.

**Table 3:** Comparison of self-management behavioral stages between the 2 groups before and after intervention examples (N=49).

Time group			Precontemplation, n (%)		Contemplation, n (%)		Preparatory, n (%)		Action, n (%)		Maintenance, n (%)		Wald chi-square (*df*)^a^			*P* value^a^			Odds ratio (95%CI)^a^	
**Preintervention**														0.176 (1)			.68			1.179 (0.546-2.546)
	Control group	25 (51.02)		7 (14.29)		4 (8.16)		9 (18.37)		4 (8.16)										
	Experimental group	22 (44.90)		9 (18.37)		3 (6.12)		10 (20.41)		5 (10.20)										
**Postintervention**														6.024 (1)			.01			2.543 (1.207-5.357)
	Control group	12 (24.49)		13 (26.53)		8 (16.33)		9 (18.37)		7 (14.29)										
	Experimental group	6 (12.24)		8 (16.33)		5 (10.20)		16 (32.65)		14 (28.57)										

^a^Ordinal logistic model was adjusted for gender, age, only-child status, disease duration, caregiver type, caregiver’s education level, residence location, and monthly household income. The Wilcoxon signed-rank test revealed a statistically significant difference in the experimental group between baseline and the 6-month postintervention assessment (Z=5.443, *P*<.001). The control group showed statistically significant differences between baseline and the 6-month postintervention assessment (Z=5.000, *P*<.001).

### Comparison of Transition Readiness Between the 2 Groups Before and After the Intervention

There was no statistical difference between the 2 groups in terms of total transition readiness scores and scores on the dimensions before the intervention (*P*>.05). At the end of 6 months of intervention, the intervention group demonstrated significantly higher scores than the control group in health care engagement (mean difference=2.28, 95% CI 0.57-3.99), disease knowledge (1.16, 95% CI 0.40-1.93), provider communication (2.08, 95% CI 0.77-3.40), transition readiness score (7.96, 95% CI 3.54-12.37), and effect sizes (Cohen *d*) ranged from 0.571 to 0.772. Both groups showed significant improvements from baseline in medication management, health care participation, disease knowledge, doctor-patient communication, and transition readiness total scores at 6-month follow-up (all *P*<.05). Notably, the intervention group achieved additional incremental benefits versus controls (medication management: 3.81, 95% CI 1.26-6.36; health care engagement: 2.77, 95% CI 0.52-5.02; disease knowledge: 1.30, 95% CI 0.28-2.31; provider communication: 3.42, 95% CI 1.62-5.22; transition readiness: 11.30, 95% CI 5.70-6.89; effect sizes [Cohen *d*] ranged from 0.527 to 0.864, indicating moderate-to-large clinical effects, as shown in Table 4).

**Table 4:** Comparison of transition readiness between the 2 groups of patients before and after the intervention.

Time and group		Medication management	Health care engagement	Diseases knowledge	Provider communication	Transition readiness score
**Preintervention**						
	Control group (n=52), mean (SD)	20.59 (6.23)	11.33 (3.63)	5.78 (2.19)	15.29 (3.52)	52.98 (9.89)
	Experimental group (n=52), mean (SD)	19.51 (5.47)	11.45 (5.02)	5.76 (1.94)	14.43 (4.40)	51.14 (13.79)
	Between-group differences, 95% CI^a^	–1.38 (–3.86 to 1.10)	–0.49 (–2.39 to 1.40)	–0.13 (–1.02 to 0.75)	–1.33 (–3.05 to 0.39)	–3.34 (–8.67 to 2.00)
	t^a^	–1.108	–0.518	–0.294	–1.542	–1.244
	*P* value^a^	.271	.606	.769	.127	.217
**Postintervention**						
	Control group (n=49), mean (SD)	22.80 (6.18)	13.33 (3.80)	7.35 (1.75)	16.02 (3.14)	59.49 (9.99)
	Experimental group (n=49), mean (SD)	25.31 (5.32)	16.00 (4.12)	8.57 (1.80)	18.47 (3.08)	68.35 (10.51)
	Between-group differences, 95% CI^a^	2.43 (–0.03 to 4.89)	2.28 (0.57 to 3.99)	1.16 (0.40 to 1.93)	2.08 (0.77 to 3.40)	7.96 (3.54 to 12.37)
	t^a^	1.963	2.653	3.021	3.152	3.587
	*P* value^a^	.053	.010	.003	.002	.001
	Cohen *d*^a^	0.422	0.571	0.650	0.678	0.772
**Postintervention changes**						
	Control group (n=49), mean (SD)	2.20 (4.99)^b^	2.00 (2.45)^b^	1.57 (2.25)^b^	0.73 (2.35)^b^	6.51 (7.89)^b^
	Experimental group (n=49), mean (SD)	5.80 (6.28)^b^	4.55 (6.57)^b^	2.82 (2.55)^b^	4.04 (5.44)^b^	17.20 (15.78)^b^
	Between-group differences, 95% CI^a^	3.81 (1.26-6.36)	2.77 (0.52-5.02)	1.30 (0.28-2.31)	3.42 (1.62-5.22)	11.30 (5.70-16.89)
	t^a^	2.973	2.452	2.533	3.780	4.018
	*P* value^a^	.004	.016	.013	<.001	<.001
	Cohen *d*^a^	0.639	0.527	0.545	0.813	0.864

^a^Analysis of covariance (ANCOVA) was performed with adjustments for gender, age, only-child status, disease duration, caregiver type, caregiver’s education level, residence, and monthly household income.

^b^The paired *t* test showed a statistically significant result (*P*<.05)

### Evaluation of the Acceptability of the Intervention Program in the Experimental Group

Adolescents with epilepsy rated the program as low as “This approach solves some of my emotional problems” (3.45, SD 0.82 points), and medical staff rated it as low as “This approach solves personalized problems” (3.70, SD 0.48 points). The overall satisfaction scores were 4.43 (SD 0.50) for patients and 4.16 (SD 0.82) for health care providers, as shown in Table 5.

**Table 5:** Acceptance ratings of transtheoretical model–based mobile health (mHealth) management program.

Item title			Score, mean (SD)
**Evaluation of the program by adolescents with epilepsy**			
	1. I like this remote group intervention approach	4.43 (0.79)	
	2. Appropriate frequency of interventions	3.96 (0.71)	
	3. Parental involvement is helpful to me	3.67 (0.69)	
	4. The words and actions of other patients in the discussion had an impact on me	3.94 (0.77)	
	5. This approach allows for normal communication	4.02 (0.69)	
	6. This way I can learn about diseases	4.43 (0.65)	
	7. This approach teaches me self-management	4.16 (0.59)	
	8. This approach can solve some of my emotional problems.	3.45 (0.82)	
	9. This approach saves time and energy	4.16 (0.72)	
	10. This approach saves spending on travelling to the site	4.63 (0.49)	
	11. I think it could be promoted to other young people with epilepsy who need it	4.18 (0.57)	
	12. General feeling of satisfaction	4.43 (0.50)	
**Evaluation of the program by medical staff**			
	1. This approach helps patients	4.60 (0.52)	
	2. This approach allows for normal communication with the patient.	4.10 (0.74)	
	3. This approach addresses personalization	3.70 (0.48)	
	4. This approach saves time and effort relative to individualized interventions	4.50 (0.53)	
	5. I think it can be used in clinical practice	4.10 (0.64)	
	6. General feeling of satisfaction	4.16 (0.82)	

## Discussion

### The TTM-Based mHealth Intervention May Facilitate the Establishment of Self-Management Behaviors in Adolescents With Epilepsy

The survey found that patients with chronic diseases have different information needs at various stages of behavior change [26]. The greatest advantage of TTM is that it recognizes the differences between individuals at different stages of behavior. Stage-matched behavior change strategies were implemented by researchers, which enhanced participation rates and promoted patients’ progression toward the action and maintenance stages [11]. At the end of 6 months of intervention, the intervention group demonstrated significantly better self-management behavior stages compared with controls (OR 2.543, 95% CI 1.207-5.357, *P*=.01). At the end of 6 months of intervention, the majority of participants in the intervention group reached the action stage (16/49, 32.65%) and maintenance stage (14/49, 28.57%), whereas most controls remained in precontemplation (12/49, 24.49%) and contemplation stages (13/49, 26.53%). Evidently, adolescent patients with epilepsy who received stage-matched behavioral interventions demonstrated favorable self-management behavior modifications, with the majority sustaining these changes as habitual practices. This study protocol used individualized strategies tailored to participants’ specific stages of behavioral change, effectively addressing unique needs while enhancing intrinsic motivation for engagement. The technology-delivered remote intervention aligned well with adolescents’ social habits, potentially improving participation rates and thereby better supporting behavioral transitions. These coordinated approaches successfully promoted the establishment of healthy self-management behaviors among youth with epilepsy, including medication adherence, regular follow-ups, and maintenance of healthy sleep routines. Furthermore, while individual behaviors represent manifestations of personal volition and psychological factors, they are profoundly influenced by external environmental contexts [27]. When patients at identical behavioral stages and their caregivers participated in group educational sessions with peer discussions, observed peer behaviors, and shared experiences these factors potentially induced social conformity effects, thereby facilitating positive behavioral modifications. The self-management applet gives patients a convenient and informative reminder service that stimulates and reinforces self-management behaviors. As self-management behaviors in the adolescent-to-adult transition with epilepsy shift to the action and retention phases, self-management skills improve accordingly, facilitating the transition of adolescents with epilepsy to adult medical care. Our findings are consistent with previous research applying the TTM to other chronic conditions. For instance, a study on diabetes self-management demonstrated that stage-matched interventions significantly improved participants’ progression to action and maintenance stages, leading to better glycemic control [28]. Similarly, research in heart failure patients showed that TTM-based counseling was effective in promoting medication adherence and healthy lifestyles [29]. This convergence of evidence across different diseases strengthens the theoretical robustness of the TTM and suggests its broad applicability in chronic disease management, including epilepsy.

Notably, the establishment of self-management behaviors in patients with chronic conditions constitutes a complex and protracted process [30]. This necessitates sustained clinical support from health care providers. Consequently, health care professionals should first acknowledge the longitudinal nature and potential challenges of behavior formation, which fosters patient understanding and trust while facilitating patient-centered support strategies. Furthermore, to promote long-term self-management behaviors among adolescents with epilepsy, clinicians can enhance self-efficacy through collaborative goal-setting, individualized education, and encouragement of self-monitoring and decision-making. Improved self-efficacy and self-management capacity exhibit bidirectional positive reinforcement [31,32], thereby contributing to sustainable health behavior establishment. Additionally, leveraging modern mHealth technologies enables convenient and continuous therapeutic alliances. These digital solutions eliminate temporal and spatial barriers, particularly engaging tech-savvy adolescents through accessible communication channels. Integrated functions such as automated monitoring, seamless communication, and reminder systems may further optimize patient engagement. Finally, family members and caregivers should be actively involved in the health management process, as their provision of emotional support and practical assistance reinforces patients’ motivation for self-management.

### The TTM-Based mHealth Intervention May Enhance Transition Readiness Among Adolescents With Epilepsy

This study found that at baseline, adolescents with epilepsy demonstrated moderate transition readiness levels, with mean scores of 50.75 (SD 14.02) (intervention group) and 53.10 (SD 9.76) (control group). At the end of 6 months of intervention. Both groups showed significant improvements from baseline in medication management, health care participation, disease knowledge, doctor-patient communication, and transition readiness total scores at 6-month follow-up (all *P*<.05). Notably, the intervention group achieved additional incremental benefits versus controls (medication management: 3.81, 95% CI 1.26-6.36; health care engagement: 2.77, 95% CI 0.52-5.02; disease knowledge: 1.30, 95% CI 0.28-2.31; provider communication: 3.42, 95% CI 1.62-5.22; transition readiness: 11.30, 95% CI 5.70-6.89; effect sizes [Cohen *d*] ranged from 0.527 to 0.864, indicating moderate-to-large clinical effects). It is evident that the TTM-based mHealth intervention may comprehensively enhance transition readiness in adolescents with epilepsy through multiple mechanisms. First, stage-matched behavioral strategies address individualized patient needs, thereby improving self-efficacy and engagement in self-management practices. This personalized approach motivates active learning of disease knowledge and self-care skills. The mobile-delivered remote intervention reduces psychological barriers by leveraging adolescents’ preference for digital communication, creating a more relaxed interaction environment compared with face-to-face encounters. The program’s exclusive access for patients (excluding parents) fosters independent disease management, while strengthened patient-provider collaboration builds problem-solving confidence through shared decision-making. During the action phase, the intervention systematically reduces parental over-management of medical tasks while establishing collaborative parent-adolescent partnerships, thereby enhancing autonomous self-management and strengthening health care participation and health responsibility. The mHealth platform sustains behavioral engagement through tailored health education delivery, medication adherence reminders, and incentivized tracking mechanisms, which collectively facilitate disease-specific knowledge acquisition, proper pharmacotherapy management, and ultimately the internalization of stable health behaviors. While a predominant focus of many existing mHealth solutions in epilepsy has been on fundamental features such as seizure tracking and medication reminders, the innovative core of our program lies in its strong theoretical foundation in the TTM, which is specifically designed to drive purposeful and progressive behavioral change. This represents a significant evolution from passive monitoring tools towards a more dynamic system of active, staged coaching. It is widely recognized that a common limitation in the field of digital health is the development of apps that provide informational support but lack a robust theoretical framework to guide behavioral modification. Our study directly addresses this gap. The results demonstrate that a theory-driven, behaviorally-focused mHealth intervention can effectively target the multifaceted challenge of transition readiness, which encompasses not only the acquisition of knowledge but also the critical development of skills, motivation, and self-efficacy. Current surveys indicate that adolescents predominantly use mobile platforms during leisure time to access science education information [33]. Mobile-delivered interventions enable precise and concise health education, allowing patients to engage in learning anytime and anywhere during fragmented free moments. This approach not only enhances participation rates but also facilitates timely identification and intervention by health care providers, ultimately supporting adolescents with epilepsy in achieving optimal transition readiness and successful transfer to adult health care systems.

The intervention demonstrated high acceptability among both adolescents with epilepsy and participating health care providers. Existing studies on the evaluation of services for the transition from chronically ill adolescents to adults have focused on indicators of care improvement, disease outcomes, quality of life, and skills improvement, with few evaluating the acceptability of the intervention program [8]. The overall acceptance of the program in this study was good. Adolescents with epilepsy rated the lowest score as “This approach can solve some of my emotional problems,” which may be related to the complexity of psychological or emotional problems in people with epilepsy and the need to intervene in multiple ways [34], suggesting that health professionals need to develop the most appropriate intervention strategies based on a comprehensive assessment, taking into account the environmental and social needs of the individual and the family, when providing psychological interventions. The most appropriate intervention strategies. For example, while common psychological problems can be addressed through efficient and cost-effective group interventions, attention needs to be paid to assessing the psychological needs of specific individuals and targeting interventions. In addition, health care professionals believe that more individualized interventions save time and effort but may be less effective in addressing individualized problems [35]. Therefore, given the limited human resources in health care, group interventions should be maximized, while taking into account the need to identify specific individual problems and individualized interventions.

### Conclusions

The TTM-based mHealth intervention demonstrated good feasibility for transitional care in adolescents with epilepsy. This program enabled personalized group interventions while making health management more accessible and continuous. The approach may facilitate healthy behavior establishment and enhance transition readiness, thereby supporting successful transfer to adult health care. Both patients and health care providers reported high acceptability, suggesting good clinical translatability.

Because allocation was by hospital district, the sample was small and self-reported, and follow-up was limited to 6 months. Furthermore, the participants were restricted to adolescents with nonrefractory epilepsy and without cognitive impairment, excluding those with more complex disease characteristics. The results should be interpreted with caution. Future studies should expand the sample size, extend the intervention and follow-up periods, and use randomized controlled trial designs to further validate the intervention’s efficacy. Additionally, research should focus on transitional care for adolescents with comorbid cognitive impairments or poorly controlled seizures. This study assessed acceptability using descriptive methods; future work should explore standardized or quantifiable evaluation approaches.

## Appendix

Multimedia Appendix 1 TREND checklist.

## Abbreviations

mHealth: mobile health
OR: odds ratio
SMD: standardized mean difference
STARx: Self-Management and Transition to Adulthood with Rx=Treatment
TTM: transtheoretical model

## References

[1] Andrade DM, Bassett AS, Bercovici E, Borlot F, Bui E, Camfield P, Clozza GQ, Cohen E, Gofine T, Graves L, Greenaway J, Guttman B, Guttman-Slater M, Hassan A, Henze M, Kaufman M, Lawless B, Lee H, Lindzon L, Lomax LB, McAndrews MP, Menna-Dack D, Minassian BA, Mulligan J, Nabbout R, Nejm T, Secco M, Sellers L, Shapiro M, Slegr M, Smith R, Szatmari P, Tao L, Vogt A, Whiting S, Carter Snead O (2017). Epilepsy: transition from pediatric to adult care. recommendations of the Ontario epilepsy implementation task force. Epilepsia.

[2] Betz CL (2017). SPN position statement: transition of pediatric patients into adult care. J Pediatr Nurs.

[3] Camfield P, Camfield C (2011). Transition to adult care for children with chronic neurological disorders. Ann Neurol.

[4] Geerlings RPJ, Aldenkamp AP, de With PHN, Zinger S, Gottmer-Welschen LMC, de Louw AJA (2015). Transition to adult medical care for adolescents with epilepsy. Epilepsy Behav.

[5] Camfield PR, Andrade D, Camfield CS, Carrizosa-Moog J, Appleton R, Baulac M, Brown L, Menachem EB, Cross H, Desguerre I, Grant C, Hosny H, Jurasek L, Mula M, Pfäfflin M, Rheims S, Ring H, Shellhaas RA, Vinayan KP, Wirrell E, Nabbout R (2019). How can transition to adult care be best orchestrated for adolescents with epilepsy?. Epilepsy Behav.

[6] Mazur A, Dembinski L, Schrier L, Hadjipanayis A, Michaud P (2017). European academy of paediatric consensus statement on successful transition from paediatric to adult care for adolescents with chronic conditions. Acta Paediatr.

[7] Fredericks EM (2017). Transition readiness assessment: the importance of the adolescent perspective. Pediatr Transplant.

[8] White PH, Cooley WC, Transitions Clinical Report Authoring Group, American Academy of Pediatrics, American Academy of Family Physicians, American College of Physicians (2018). Supporting the health care transition from adolescence to adulthood in the medical home. Pediatrics.

[9] Wagner JL, Modi AC, Johnson EK, Shegog R, Escoffery C, Bamps Y, Austin JK, Schultz RJ, MapelLentz S, Smith G (2017). Self-management interventions in pediatric epilepsy: what is the level of evidence?. Epilepsia.

[10] Chai K, Zhang Y, Chang K (2020). Regional disparity of medical resources and its effect on mortality rates in China. Front Public Health.

[11] Prochaska JO, Velicer WF (1997). The transtheoretical model of health behavior change. Am J Health Promot.

[12] Zare M, Tarighat-Esfanjani A, Rafraf M, Shaghaghi A, Asghari-Jafarabadi M, Shamshiri M (2020). The barriers and facilitators of self-management among adults with type 2 diabetes mellitus: a trans theoretical model (TTM)-based mixed method study in Iran. Diabetes Metab Syndr Obes.

[13] Teng H, Yen M, Fetzer S, Sung J, Hung S (2021). Tailoring health-promoting programs for patients with chronic kidney disease: randomized controlled trial. West J Nurs Res.

[14] Liu YS, Ren HY (2019). The application progress of trans-theoretical model on changing the healthy behavior of patients with coronary heart disease. Zhonghua Xin Xue Guan Bing Za Zhi.

[15] Cruz-Ramos NA, Alor-Hernández G, Colombo-Mendoza LO, Sánchez-Cervantes JL, Rodríguez-Mazahua L, Guarneros-Nolasco LR (2022). mHealth apps for self-management of cardiovascular diseases: a scoping review. Healthcare (Basel).

[16] Bene BA, O'Connor S, Mastellos N, Majeed A, Fadahunsi KP, O'Donoghue J (2019). Impact of mobile health applications on self-management in patients with type 2 diabetes mellitus: protocol of a systematic review. BMJ Open.

[17] Alzamanan MZ, Lim K, Akmar Ismail M, Abdul Ghani N (2021). Self-management apps for people with epilepsy: systematic analysis. JMIR Mhealth Uhealth.

[18] Khoshkangin A, Agha Seyyed Esmaeil Amiri FS, Ghaddaripouri K, Noroozi N, Mazaheri Habibi MR (2023). Investigating the role of mobile health in epilepsy management: a systematic review. J Educ Health Promot.

[19] Virella Pérez YI, Medlow S, Ho J, Steinbeck K (2019). Mobile and web-based apps that support self-management and transition in young people with chronic illness: systematic review. J Med Internet Res.

[20] Des Jarlais DC, Lyles C, Crepaz N, TREND Group (2004). Improving the reporting quality of nonrandomized evaluations of behavioral and public health interventions: the TREND statement. Am J Public Health.

[21] Fisher RS, Acevedo C, Arzimanoglou A, Bogacz A, Cross JH, Elger CE, Engel J, Forsgren L, French JA, Glynn M, Hesdorffer DC, Lee BI, Mathern GW, Moshé SL, Perucca E, Scheffer IE, Tomson T, Watanabe M, Wiebe S (2014). ILAE official report: a practical clinical definition of epilepsy. Epilepsia.

[22] Scheffer IE, Berkovic S, Capovilla G, Connolly MB, French J, Guilhoto L, Hirsch E, Jain S, Mathern GW, Moshé SL, Nordli DR, Perucca E, Tomson T, Wiebe S, Zhang Y, Zuberi SM (2017). ILAE classification of the epilepsies: position paper of the ILAE commission for classification and terminology. Epilepsia.

[23] Ferris M, Cohen S, Haberman C, Javalkar K, Massengill S, Mahan JD, Kim S, Bickford K, Cantu G, Medeiros M, Phillips A, Ferris MT, Hooper SR (2015). Self-management and transition readiness assessment: development, reliability, and factor structure of the StarX questionnaire. J Pediatr Nurs.

[24] Ma J, Yu Q, Ding W, Zhang T, Zhang Y (2021). Psychometric properties of the 'Self-Management and Transition to Adulthood with R = Treatment Questionnaire' in Chinese children and young people with chronic diseases. Int J Nurs Pract.

[25] Yamamoto K, Ito M, Sakata M, Koizumi S, Hashisako M, Sato M, Stoyanov SR, Furukawa TA (2022). Japanese version of the mobile app rating scale (MARS): development and validation. JMIR Mhealth Uhealth.

[26] Wood AR, Ross L, Wood RJ (2023). Motivational interviewing and chronic care management using the transtheoretical model of change. Health Soc Work.

[27] Subotnik RF, Olszewski-Kubilius P, Worrell FC (2019). Environmental factors and personal characteristics interact to yield high performance in domains. Front Psychol.

[28] Dunkel A, von Storch K, Hochheim M, Zank S, Polidori MC, Woopen C (2025). Long-term effects of transtheoretical model-based lifestyle intervention on self-efficacy and self-management in patients with type 2 diabetes - randomised controlled trial. Int J Behav Med.

[29] Yuan D, Xue Y, Zhou Y (2025). Improving nutritional status in chronic heart failure patients: effectiveness of a transtheoretical model-based stepwise nutritional management program. Risk Manag Healthc Policy.

[30] Ouyang W, Chen H, Xu X, Zhang X, Fu L, Tang F, Wen Z, Marrone G, Liu L, Lin J, Liu X, Wu Y (2022). Self-management program for patients with chronic kidney disease (SMP-CKD) in Southern China: protocol for an ambispective cohort study. BMC Nephrol.

[31] Wang Y, Masingboon K, Wacharasin C (2025). Mediating role of self-efficacy in the relationship between family functioning and self-management behaviors in patients with coronary heart disease: a cross-sectional study in Jiangsu, China. Belitung Nurs J.

[32] Zhang R, Li X, Luo H, Niu J, Zhang H (2025). Effect of self-efficacy, disease perception, social support, anxiety, and depression on self-management in young patients with stroke. J Neurosci Nurs.

[33] Goodyear VA, Armour KM, Wood H (2019). Young people and their engagement with health-related social media: new perspectives. Sport Educ Soc.

[34] Goselink RJM, Olsson I, Malmgren K, Reilly C (2022). Transition to adult care in epilepsy: a systematic review. Seizure.

[35] Michaelis R, Tang V, Goldstein LH, Reuber M, LaFrance WC, Lundgren T, Modi AC, Wagner JL (2018). Psychological treatments for adults and children with epilepsy: evidence-based recommendations by the international league against epilepsy psychology task force. Epilepsia.

