# Supplementary material for: Effects of a Transtheoretical Model–Based mHealth Intervention on Transition Readiness in Adolescents With Epilepsy: Quasi-Experimental Study
Source: JMIR Mhealth Uhealth. 2025 Nov 11;13:e70085. doi: 10.2196/70085 (PMC12648129; doi:10.2196/70085)
Supplement: Multimedia Appendix 1 [file mhealth_v13i1e70085_app1.docx]

**TREND Checklist**

Transparent Reporting of Evaluations with Nonrandomized Designs

Manuscript Title: **[Effects of a Transtheoretical Model-Based mHealth Intervention on Transition Readiness in Adolescents with Epilepsy: A Quasi-Experimental Study]**

Corresponding Author: [Huaying Yin]

Checklist Completed by: [Qing Xia]

Instructions: Please indicate the page number(s) where the item is reported. If an item is not applicable to your study, please write "N/A" and provide a brief explanation.

| No. | Item | Page Number(s) | Description from Your Manuscript |
| --- | --- | --- | --- |
| 1 | Intervention  a. Rationale or background for the intervention and its components. | 4 | Introduction: Background on TTM and mHealth for reducing costs and personalizing care in epilepsy transition services. |
|  | b. Eligibility criteria for participants, providers, and settings. | 6 | Objects of study: Inclusion: 12-18 y/o, non-refractory epilepsy, seizure-free >30 days. Exclusion: smartphone impairment, communication disorders, comorbidities. |
| 2 | Theoretical model  Description of the theoretical model used, if applicable. | 4 | Introduction & Methods: Transtheoretical Model (TTM) is described as the foundation for the stage-tailored intervention. |
| 3 | Research question  Statement of the research question or hypothesis. | 2 | Objective: "This quasi-experimental study evaluates the feasibility of TTM-based mHealth management for improving transition services..." |
| 4 | Outcomes  a. Clearly defined primary and secondary outcome measures. | 7 | Evaluation indicators: Self-management Stage, STARx (medication management, engagement, knowledge, communication, total score), Acceptability questionnaire. |
|  | b. Methods used to collect data and any methods used to enhance the quality of measurements. | 7 | Clinical data collection: On-site and Questionnaire Star. Data checked by both researchers. |
| 5 | Assignment method  a. Unit of assignment (e.g., individual, group, community). | 1,5 | Methods & Objects of study: Individual participants were allocated by cluster (treatment location: new vs. old campus). |
|  | b. Method of assignment and the reasons for choosing that method. | 1 | Objects of study: "To avoid contamination between study groups, participants... were assigned to the intervention group, while those from the old campus served as the control group." |
| 6 | Blinding (Masking)  Whether participants, providers, and outcome assessors were blinded to the assignment. | N/A | Not blinded due to the nature of the behavioral intervention. This is a common limitation in such studies. |
| 7 | Sample size  a. How and when the sample size was determined. | 6 | Sample size calculation: Formula provided, parameters (α=0.05, power=0.9, δ=5, σ=7), calculated n=47/group accounting for dropout. |
| 8 | Statistical methods  a. Methods used to compare outcomes for each group. | 10 | Statistical methods: t-tests, ANCOVA (for between-group differences, 95% CI, Cohen's d), Chi-square, Mann-Whitney U, ordinal logistic regression, Wilcoxon signed-rank. |
| 9 | Participant flow  A flow diagram. | 19 | Note: A CONSORT-style participant flow diagram (Figure 1) illustrates the processes of recruitment, allocation, follow-up, and analysis. |
| 10 | Recruitment  Dates defining the periods of recruitment and follow-up. | 6 | Objects of study: "between August 2021 and February 2022". Methods: "The total intervention duration was six months". |
| 11 | Baseline data  A table showing baseline demographics and clinical characteristics for each group. | 10 | Statistical methods: "Baseline balance between... groups was assessed using standardized mean differences (SMD)". (Implies a baseline table exists, ensure it is presented in results). |
| 12 | Outcomes  a. For each primary and secondary outcome, results for each group. | 10,11 | Results: Self-management stages, STARx domain scores, and acceptability scores are reported for both groups. |
|  | b. Report of absolute and relative effect sizes is recommended. | 10,11 | Results: Between-group differences (absolute effect) with 95% CI and Cohen's d (standardized effect size) are reported. |
| 13 | Summary of results  a. Summary of results, based on analysis. | 10,11 | Results & Conclusion: The intervention group showed significant improvements in outcomes compared to controls. |
| 14 | Ancillary analyses  a. Report of any other analyses performed. | 10 | Statistical methods: Within-group pre-post changes were assessed using the Wilcoxon signed-rank test. |
| 15 | Adverse events  All important adverse events related to the intervention. | N/A | No adverse events related to the mHealth intervention were reported or monitored |
| 16 | Interpretation  a. Interpretation of the results, considering study hypotheses. | 14,15 | Conclusion: The program may effectively improve outcomes and facilitate transition. Findings need confirmation by larger RCTs. |
| 17 | Generalizability  Generalizability (external validity) of the trial findings. | 14,15 | Conclusion: "this study was a single-center... trial with a small sample size", limiting generalizability. |
| 18 | Evidence  General interpretation of the results in the context of current evidence. | 14,15 | Conclusion: The program provides a reference for clinical protocols. Results are consistent with positive findings of TTM and mHealth in other fields. |
